# Contagious Crying Revisited: A Cross‐Cultural Investigation Into Infant Emotion Contagion Using Infrared Thermal Imaging

**DOI:** 10.1111/desc.13608

**Published:** 2025-01-14

**Authors:** C. Vreden, E. Renner, H. E. Ainamani, R. Crowther, B. Forward, S. Mazari, G. Tuohy, E. Ndyareeba, Zanna Clay

**Affiliations:** ^1^ Department of Psychology Durham University Durham UK; ^2^ Department of Psychology Northumbria University Newcastle upon Tyne UK; ^3^ Department of Mental Health Kabale University Kabale Uganda; ^4^ Department of Foundations of Education Kabale University Kabale Uganda

**Keywords:** cross‐cultural, emotional contagion, empathy, infrared thermal imaging

## Abstract

Contagious crying in infants has been considered an early marker of their sensitivity to others’ emotions, a form of emotional contagion, and an early basis for empathy. However, it remains unclear whether infant distress in response to peer distress is due to the emotional content of crying or acoustically aversive properties of crying. Additionally, research remains severely biased towards samples from Europe and North America. In this study, we address both aspects by employing the novel and non‐invasive method of infrared thermal imaging, in combination with behavioural markers of emotional contagion, to measure emotional arousal during a contagious crying paradigm in a cross‐cultural sample of 10‐ to 11‐month‐old infants from rural and urban Uganda and the United Kingdom (*N* = 313). Infants heard social stimuli of positive, negative, and neutral emotional valence (infant laughing, crying, and babbling, respectively) and a non‐social, acoustically matched artificial aversive sound. Results revealed that overall changes (as opposed to positive or negative) in infant nasal temperature were larger in response to crying and laughing compared to the artificial aversive sound and larger for crying than for babbling. Infants showed stronger behavioural responses for crying than for the artificial stimulus, as well as for crying than for laughing. Overall, our results support the view that infants within the first year of life experience emotional contagion in response to peer distress, an effect that is not just explained by the aversive nature of the stimuli. Sensitivity to others’ emotional signals in the first year of life may provide the core building blocks for empathy.

## 1 Introduction

A key component of empathy is the ability to share or experience the emotions of another individual (de Waal 2007; Eisenberg et al. 2006). The core underlying mechanism for this is emotional contagion, that is, the transfer of emotional states from one individual to another. Evidence of emotional contagion has typically been based on a phenomenon known as “contagious crying” in which the distressed cries of one infant trigger distress in other infants. Studies of contagious crying have a long‐standing history in emotional development research, with Simner (1971) being one of the first to empirically document it in neonates: 3‐day‐old infants showed the highest levels of contagious crying in response to a similarly aged infant's cry as compared to white noise, an artificial cry, or an older infant's cry. Evidence of this phenomenon during the first days of life has since been replicated numerous times (Dondi, Simion, and Caltran 1999; Martin and Clark 1982; Sagi and Hoffman 1976).

**Summary**

- Contagious crying has been proposed as an early marker of emotional sensitivity and empathy in infancy; however, questions remain as to what extent it is motivated by emotional arousal versus acoustic aversiveness.
- Using the emerging method of infra‐red thermal imaging, we investigated the development of emotional contagion in a cross‐cultural sample of over 300 infants from Uganda and United Kingdom.
- This is the first study to apply infrared thermal imaging to study infant emotional development in a non‐laboratory context in sub‐Saharan Africa.
- Results support the presence of physiological and behavioural emotional contagion in the first year of life, a core basis for empathy.

Despite being used as an established measure of infant emotional contagion for multiple decades, more recent work has challenged assumptions about the empathic nature of contagious crying. Ruffman, Lorimer, and Scarf (2017) argued that rather than emotional contagion, contagious crying could be a reaction to acoustically aversive features or stimulus novelty. Previous studies have used control conditions featuring silence or white noise, which do not have the same aversive acoustic features as crying. Moreover, previous work has often used only one cry stimulus, making it hard to decipher if the acoustic properties of that cry (and not its negative emotional content) result in infant negative responses. Considering that at least adults are sensitive to acoustic variation in infant crying and can link it to different levels of distress intensity (Esposito et al. 2015), and that even non‐social aversive sounds, such as fingernails on a blackboard, can elicit emotional reactions (Reuter and Oehler 2011), the selection of appropriate stimuli and controls is especially important. An additional limitation of previous contagious crying research with regards to stimuli selection is the use of long and intense distress stimuli. Earlier studies used distress vocalisations of 4 to 6 min (e.g., Dondi, Simion, and Caltran 1999; Simner 1971). In these studies, participant distress was frequent but only set in after 2 to 3 min. In studies with shorter distress stimuli (for example, 60 s; Roth‐Hanania, Davidov, and Zahn‐Waxler 2011), participants became distressed very rarely, most likely due to the shorter stimuli being more appropriate for the emotion regulation capacities of infants.

Ruffman et al. (2019) addressed some of these methodological issues by comparing the behavioural response (happiness vs. sadness) of adults and 2‐year‐old toddlers to videos of an infant crying, laughing, and babbling, and a video of a neutral infant, overlaid with white noise matched to the acoustic properties of crying. Toddler responses were significantly more positive, measured by happier facial expressions, in response to laughing than to any of the other stimuli, indicative of emotional contagion in response to positive social stimuli. However, responses to crying and white noise were similar, with a slightly negative behavioural response to both. This study calls into question whether the observed distress response was a sign of emotional contagion or a reaction to aversive acoustic features of the crying and white noise stimuli. Interestingly, toddlers also showed no difference in behavioural responses between babbling and crying. Stimuli in this study were 1 min long, which may have contributed to the low reactivity of toddlers, as at this length they were likely able to regulate their own emotional arousal and thus not express self‐distress. Additionally, as described further below, the lack of emotional contagion on a behavioural level should not be taken as a lack of emotional contagion overall, as shared affective states can occur on a physiological level, even without behavioural affect matching (Preston and Hofelich 2012). For example, in adults, imagining and observing others in pain and imagining oneself or another experiencing fear or anger leads to similar neural activation (Decety and Jackson 2006; Preston et al. 2007). Similarly, adults watching another adult completing stressful tasks leads to increased stress hormone levels in the observer (Buchanan et al. 2012). Research on physiological manifestations of emotional contagion in early development is more limited but shows comparable results: For example, infants show greater pupil dilation in response to peer‐positive and negative compared to neutral emotional states (Geangu et al. 2011).

A novel way of studying physiological emotional arousal is infrared thermal imaging, a non‐invasive, contact‐free method of capturing changes in surface skin temperature in response to arousing situations (Ioannou, Gallese, and Merla 2014). Autonomic activation due to emotional arousal leads to changes in subcutaneous blood flow, which can be measured on the skin surface, providing a close to real‐time measure of physiological affective responses (Travain and Valsecchi 2021). Facial thermal changes have been observed for both experiencing positive and negative emotional states (e.g., Aureli et al. 2015; Ioannou et al. 2013; Ioannou, Gallese, and Merla 2014) as well as in response to others’ states: Adults crying sympathetically in response to emotionally arousing movies showed an increase in nose tip temperature relative to baseline (Ioannou et al. 2016). Mothers observing their 3‐year‐old children in distress showed similar thermal patterns as their child (Ebisch et al. 2012), indicative of emotional contagion. Similar thermal imaging results also come from captive and wild primates, whose skin temperature changes in response to witnessing emotionally arousing social situations, like hearing conspecific social conflicts or distress (Dezecache et al. 2017; Kano et al. 2016). With its flexibility to be employed across a wide array of contexts, thermal imaging thus provides a novel way to study the physiological basis of emotional contagion in infants, beyond purely behavioural responses, which cannot easily detect underlying mechanisms.

Another limitation to our current understanding of the development of emotional contagion is a persistent bias towards industrialised sample populations, particularly those from Europe and North America. To our knowledge, there have been no studies of contagious crying outside of typically evaluated Western populations. This is reflective of a widespread issue in developmental psychology, with over 90% of studies published in developmental journals sampling US American, European, and/or English‐speaking participants (Nielsen et al. 2017). Despite the overwhelming over‐representation of such samples, these are not globally or historically representative of most infants’ rearing environments. Thus, any claims about universal patterns of early development should be questioned in their universality, centrality, and validity (Kline, Shamsudheen, and Broesch 2018). Although physiological emotional contagion as an automatic response may be less affected by socio‐cultural influences in early development, behavioural responses to others’ affective states appear to be shaped by different socialisation strategies for emotion expression and regulation (e.g., Keller and Otto 2009). Mothers from prototypical relational socio‐cultural settings in rural Africa, where interpersonal responsibility and group harmony are emphasised more than in the West, have been shown to prefer emotionally inexpressive infants (Keller and Otto 2009). Socialisation towards this aim may thus encourage more mature emotion regulation and less negative emotional reactivity (Bozicevic et al. 2016). To what extent variation in socialisation of emotional responding and regulation affects initial physiological emotional arousal and/or behavioural emotional contagion is an important line of research requiring a culturally diverse perspective.

In the present study, we aimed to address these constraints by conducting a systematic cross‐cultural investigation into early emotional contagion in infants through a combination of behavioural and physiological measures. Sampling from both rural and urban Uganda as well as an urban setting in the United Kingdom, we compared the thermal and behavioural responses of 10‐ to 11‐month‐old infants to different acoustic stimuli of varying emotional valence (laughing, crying, babbling), as well as a non‐social artificial aversive stimulus. The end of the first year of life is a particularly interesting age to study emotional contagion, as infants at this age respond to others’ distress with both empathic concern and self‐distress, but overt empathic prosocial behaviours are either rare or absent at this age (e.g., Davidov et al. 2021; Roth‐Hanania, Davidov, and Zahn‐Waxler 2011), making it a key timepoint in emotional development just before the onset of active empathic responding. In Uganda, due to the interplay of urbanisation and globalisation, an urban‐rural comparison provided the opportunity to compare more traditional lifestyles and caregiving practices in the rural site to those who are more exposed to Western influences (due to increased access to formal education and Western media) in the urban site. In an urbanised society like the United Kingdom, the nuance between rural and urban is less clearly distinguishable, especially with more equal access to education and media and greater mobility between urban and rural locations. Thus, differences may be less meaningful, at least for our research questions, and thus the rural‐urban comparison was not made in the United Kingdom. To address previous methodological issues, we carefully considered the intensity of acoustic stimuli to ensure they were not overly arousing for infants and also designed control stimuli that acoustically matched the features of infant crying. This enabled a more accurate assessment of whether infant responses to crying were due to its emotional features or its aversive properties.

We investigated the following research questions: Do infants experience genuine emotional contagion that cannot be explained by acoustic aversiveness? And does this contagion manifest behaviourally, as well as physiologically, as an effect of emotional arousal? Lastly, we wanted to examine how emotional contagion may be influenced by different cultural and caregiving practices, which—due to different exposure to emotional expressions and socialisation of emotion regulation—may equip infants with different ways of responding to the emotions of others. We hypothesised that, across all sites, 10‐ to 11‐month‐old infants would show emotional contagion, on both behavioural and physiological levels, for positive and negative compared to neutral social stimuli (Geangu et al. 2010; Geangu et al. 2011), as evidenced by greater affect matching (e.g., facial distress in response to crying) and greater thermal changes in nasal temperature. Additionally, although behavioural responses to negative social stimuli and a non‐social artificial aversive stimulus may be similar (Ruffman et al. 2019), we predicted greater thermal changes for the social negative stimulus (crying) than to a non‐social artificial aversive one, demonstrating evidence of emotional contagion, rather than just a reaction to acoustic aversiveness. As previous research has shown both increases and decreases in temperature in response to emotional stimuli (e.g., Ioannou et al. 2013, 2016; Nakanishi and Imai‐Matsumura 2008), our hypotheses are based on the magnitude of temperature change, rather than direction. We therefore entered raw thermal changes into our analyses so as not to miss potential variation in directionality of the change. We also predicted that behavioural markers of emotional contagion (e.g., emotionally matched facial expressions or vocalisations) would differ across sites such that Ugandan infants, whose mothers’ socialisation goals align with a preference for emotionally inexpressive infants, would be less behaviourally reactive than UK infants, whose mothers have more of a preference for autonomous expressivity (Holden et al. 2022). We did not expect the same difference for physiological emotional arousal, as this is considered an automatically activated process (Prochazkova and Kret 2017) and is thus unlikely to be influenced by socio‐cultural factors. Lastly, because prior research using thermal imaging to study emotions in infants has only measured thermography in parallel with behaviour but not directly linked the two, we explored their potential direct link. At group level, we expected results to follow the same pattern, but whether they would do so at the individual level was an exploratory question.

## 2 Methods

### 2.1 Participants and Research Sites

The present study was part of a larger longitudinal project over the first 2 years of life in three sites in Uganda and the United Kingdom. The current study was conducted when participants were between 10 and 11 months old, which was the second testing session in rural Uganda and the United Kingdom and the first session in urban Uganda.

#### 2.1.1 Mbarara

In Mbarara, a major city in Uganda, 203 infants (95 female, 108 male) participated (*M*_age (weeks)_ = 45.51, SD = 2.54; 10.47 months). To be able to detect fine‐grained drivers of variation, we recruited a larger sample at this site. Due to timeline, recruitment, and feasibility constraints, a similar sample size maximisation was not possible at the rural site. Recruitment was organised with the help of village health teams and conducted by local researchers. Mothers and infants visited the testing location in a primary school in the city.

#### 2.1.2 Budongo (Uganda)

Fifty‐three infants (28 female, 25 male) from Budongo participated in this study (*M*_age (weeks)_ = 48.36, SD = 2.98; 11.13 months). Budongo is a rural region in Western Uganda on the fringes of the Budongo forest. Participants were recruited via word of mouth by local researchers in villages in the area. Testing took place in a quiet room on a nearby college campus.

#### 2.1.3 United Kingdom

The UK sample consisted of 57 infants (31 female, 26 male) from an urban region in Northeast England (*M*_age (weeks)_ = 46.84, SD = 2.26; 10.78 months). They were recruited through online adverts on social media and local baby playgroups. Testing took place in on‐campus university laboratories. Table 1 presents further demographic characteristics of the different sites.

**TABLE 1:** Demographic information by site.

	Budongo (Rural Uganda)	Mbarara (Urban Uganda)	United Kingdom
% Completed secondary school	8.93 (37.5 completed primary school)	36.62 (66.67 completed primary school)	100
% Employed	16.07	64.79	90.63
Mean household size	5.48	4.28	3.66
% Mother acts as primary caregiver	51.79	75.11	87.5
Mean number of children in household (including participant)	3.26	2.25	1.67
Mean household income	155,000 Ugandan Shilling	1,250,000 Ugandan Shilling	£3750–5000

*Note*: Because monthly income in the United Kingdom was recorded in income bands, this number represents the median band. Employment data and income figures are pre‐maternity leave.

### 2.2 Ethics

Ethical approvals were obtained from the Department of Psychology Ethics Committees at Durham University, the Ugandan Virus Research Institute Regional Ethics Committee (GC/127/20/08/788), and the Ugandan National Council for Science and Technology (SS596ES). Due to low levels of literacy in Uganda, especially in the rural Ugandan sample, local researchers read out the information sheet and consent form in a local language where needed, and mothers who could not write consented verbally, accompanied with a thumb print, which is standard procedure for signatures in Uganda. In the United Kingdom, written informed consent was obtained through an online platform.

### 2.3 Stimuli

Each trial began with a 10 s calming cartoon sequence (from the “Moomins” TV series) without sound to attract the infant's attention to the screen. This was followed by a 60 s video of bubbles rising on a blue background (to keep the infant facing the screen and the thermal camera) overlaid with the respective audio stimulus for that condition. There were three social conditions: infant laughing for the positive valence condition, infant crying in the negative valence condition, and infant babbling in the neutral valence condition. In the non‐social artificial aversive condition, we used artificial siren sounds edited to acoustically match the amplitude variation pattern of the infant crying stimuli. We prepared four different exemplars for each condition, allowing us to determine whether infants’ responses were to a particular individual's vocal expressions or general categories of vocal expression.

The audio stimuli were obtained from online sources (see Supporting Information S1 for source URLs) or, following informed consent, from infants of individuals known to the researchers and from another researcher. The stimuli were obtained from European (United Kingdom, France, Austria) and North American infants known to the researchers. Online sources were from an English‐speaking country. However, we did pilot the ecological validity of the stimuli with United Kingdom and Ugandan members of the research team, as well as by United Kingdom and Ugandan individuals naïve to the hypothesis of the study. We normalised the audio clip volume to have the same peak amplitude across stimuli, and recordings were looped to make all stimuli 60 s long, using the free audio editing software Audacity (version 3.0.0; Audacity Team 2021). After stimulus presentation, we showed another 30 s “Moomin” clip overlaid with an instrumental lullaby to help the infant return to an emotionally neutral state.

### 2.4 Setup and Materials

**FIGURE 1:**
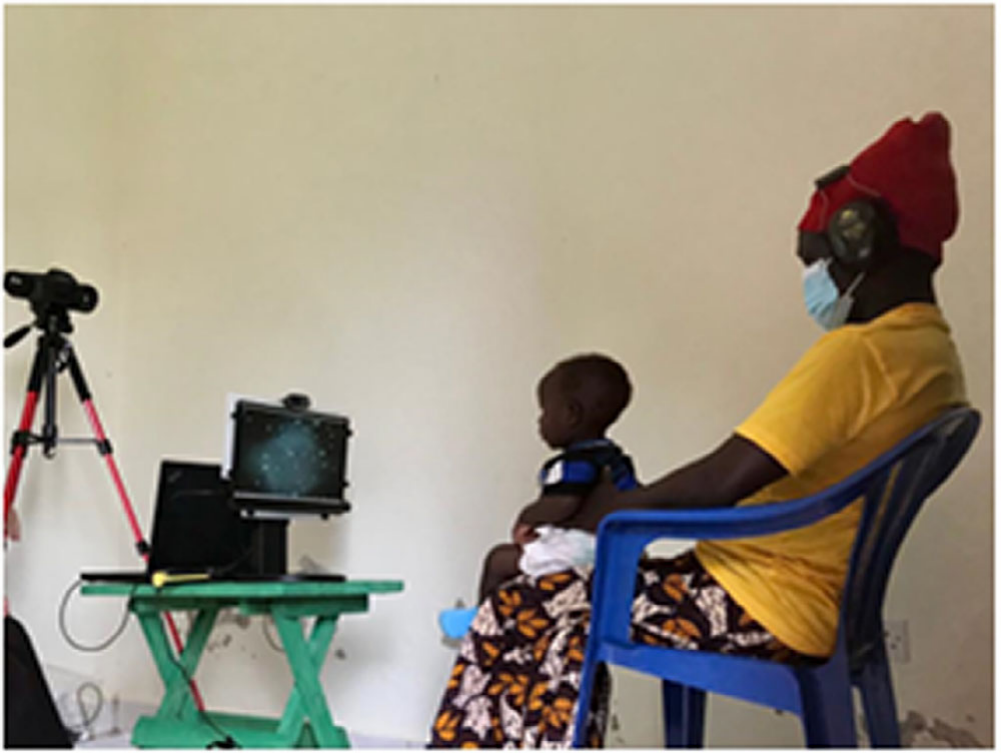
Experimental setup of the playback experiment featuring thermal camera and monitor. Images with permission.

**FIGURE 2:**
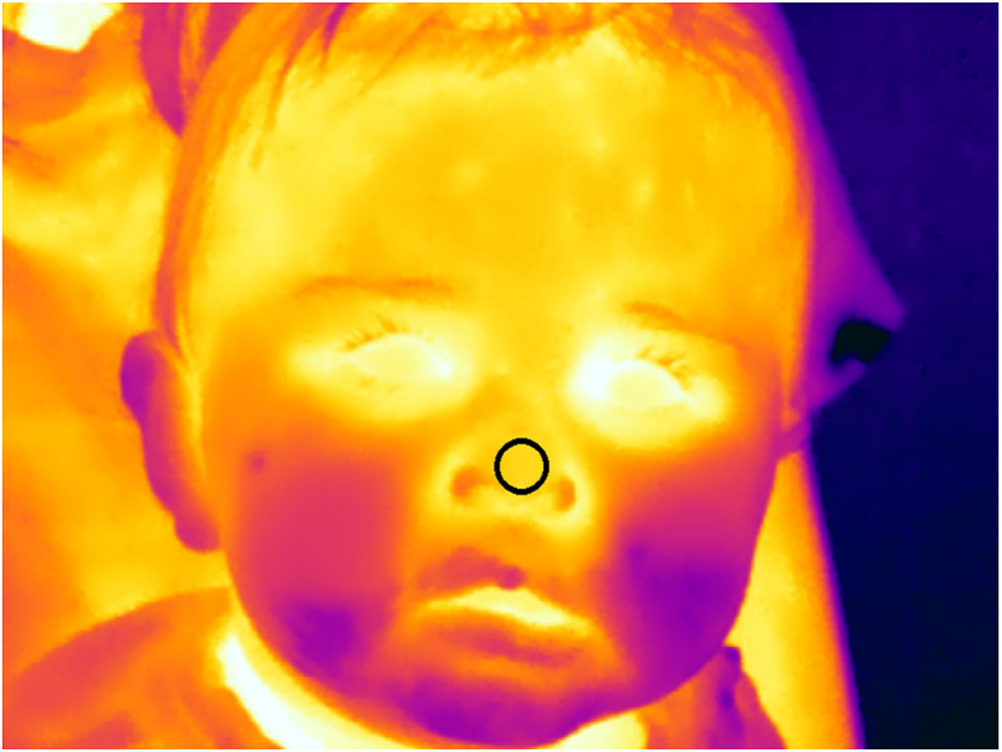
Thermal recording of the infant's face and region of interest (circle at nose tip) for temperature extraction.

Stimuli were presented on an external monitor (ASUS ZenScreen Go MB16AHP), connected to a laptop. These were set on a small table behind which the experimenter controlled the laptop and thermal camera. The infant sat on their mother's lap on a chair ca. 60 cm from the monitor (see Figure 1). Mothers’ ears were covered to avoid maternal responses to the audio stimuli being transferred to the infant. Thermal responses were filmed using an infrared thermal imaging camera (FLIR T530 with a 29 mm lens and FLIR T650 with a 24.6 mm lens) mounted on a tripod placed behind the monitor. The cameras were placed so that the participant's face was fully in frame and as close as possible to obtain comparable frame width and resolution, resulting in a difference from the participant of between 80 and 100 cm, depending on lens specifications. The two different cameras were of comparable accuracy; both had at least 320 × 270 sensor resolution and < 40 mK sensitivity (the smallest temperature difference measurable, in millikelvin). The focus of the thermal recording was on the infant's face (see Figure 2) and was adjusted manually throughout the trial. Additionally, behavioural responses were filmed using a webcam (Logitech C310 HD Webcam) at the top of the monitor. Room temperature was measured before each trial to be able to control for its effect on thermal responses (TFA KlimaLogg Pro/Infurider YF‐881D).

### 2.5 Procedure

Participants acclimatised to the temperature and humidity conditions of the testing room for at least 10–15 min beforehand (Ring and Ammer 2006), while completing other experiments (not reported in this study). These experiments included two eye‐tracking tasks, neither of which contained stimuli which could elicit strong emotional arousal responses. Both experiments were completed with the infant sitting on the mother's lap, so a carry‐over effect of facial temperature changes either due to internal states or movement was unlikely. A short break between the previous experiments and beginning of the thermal experiment of ca. 5 min while the experimenter explained the next steps provided additional time for any effects of the previous tasks to diminish. Once the infant was in a calm behavioural state and settled comfortably on their mother's lap, the trial began.

**FIGURE 3:**
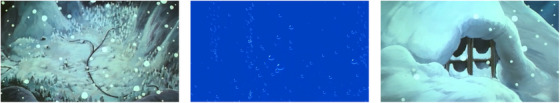
Playback trial sequence featuring baseline, stimulus, and recovery phase. Baseline showing cartoon without sound (10 s); stimulus presentation with moving bubbles background and audio overlay (60 s); recovery showing cartoon overlaid with calming instrumental music (30 s).

Each trial consisted of three phases (see Figure 3)—baseline (10 s), stimulus (60 s), and recovery (30 s)—a procedure commonly used in thermal imaging (e.g., Kano et al. 2016; Ioannou et al. 2016). During piloting, 60 s proved to be the maximum stimulus length that most infants could comfortably tolerate without becoming aggravated. After the trial, participants were given a break of at least 2 min, to allow recovery from the emotional state possibly induced by the stimulus presentation. During the break, the infant was allowed to move around the room freely. This was included following piloting, which showed infants often became distressed if held on their mother's lap for too long. The second trial was started once the participant was sat on their mother's lap again and had been sitting with minimal movement and no overt emotional behaviours for at least 1 min, so that neither physical activity during the break or their observable emotional state could affect the measurement.

All participants completed two trials based on piloting revealing that this was an acceptable number to most infants. All infants heard one negative valence (i.e., crying) trial and one other condition, with approximately equal numbers of infants hearing the babbling, laughing, and non‐social aversive stimuli. Whether the cry stimulus was presented first or second was counterbalanced across participants.

### 2.6 Facial Affect Coding

We designed a behavioural coding scheme (see Supporting Information S2) to assess facial affect, with behavioural coding completed in the open‐source video annotation tool ELAN (MPI for Psycholinguistics, https://archive.mpi.nl/tla/elan; Wittenburg et al. 2006). Negative affect coding assessed negative facial expressions, like frowning, and distress vocalisations, such as whimpering or crying. Positive affect coding assessed positive facial expressions, like smiling, and positive vocalisations, including giggling and laughing.

To compare responses to emotionally versus acoustically negative stimuli, we coded negative facial affect in response to the crying and artificial sound trials. Additionally, to compare rates of emotional contagion between positively and negatively valenced (i.e., crying) emotional stimuli, we also coded positive affect for the laughing condition. We did not code for positive affect in the crying condition, based on no observations of it during piloting. Each measure was rated on a 0–3 scale, with 0 signifying no affect and 3 high‐intensity affect. We split each 60 s trial into 4 timebins of 15 s, first coded separately and then collapsed into a mean trial score. Because infant affect may develop in a non‐linear way across the trial (e.g., sudden burst of distress or laughter), we opted for this method of averaging across the trial to get a broader measure of the infant's behavioural reactivity. The same was not applied to thermal measurements as these can be expected to develop in a more linear fashion and because we were interested in whether the valence of the emotion would lead to faster or slower temperature changes. The baseline and recovery periods of the trial were not included in behavioural coding. We conducted inter‐rater reliability for 20% of all behavioural coding with two coders. Reliability between two coders was good, with Cohen's kappa at 0.81 for positive affect and 0.78 for negative affect.

### 2.7 Thermal Data Extraction and Preparation

We extracted thermal measurements using FLIR Tools software. We measured temperatures from the nose tip which has proven to be a reliable region of interest (ROI) in previous thermal imaging studies (Ioannou et al. 2013, see Figure 2). Although some previous studies have used average ROI temperatures (e.g., Chotard, Ioannou, and Davila‐Ross 2018), we chose to use the minimal temperature. This method was chosen because both based on pilot extraction of our data and visual assessment of minimum versus average temperature changes when moving the ROI, as well as based on work by Kano et al. (2016) who report similar improved stability of the minimum temperature. Temperatures were extracted every 5 s (based on Ioannou, Gallese, and Merla 2014; Ioannou et al. 2016), with the first measurement being extracted at 0 s into the baseline and then continuously until the end of the recovery period, resulting optimally in 21 measurements per trial (3 during the baseline, 12 during the stimulus, 6 during the recovery). If the ROI was not visible, for example because the infant's head was turned or something was obscuring their nose, we checked for a better frame within 1s before or after the ideal measurement time. Visibility was considered insufficient if the pre‐defined circular region of interest which was always the same size across trials and participants could not be placed without it also covering areas outside the nose tip (e.g., nose bridge or nostrils). Measurements with unexpected abrupt changes in temperature were excluded as we suspected artifacts, such as saliva on the infant's face. Based on visual inspection of the raw data, changes were considered abrupt if the recorded temperature differed from the measurement 5 s prior by more than 0.5°.

We conducted inter‐coder reliability on all thermal measurements using intraclass correlation coefficients for 20% of the thermal data between three coders. We found very good reliability with intraclass correlation coefficients of 0.93–0.96.

### 2.8 Statistical Analysis

#### 2.8.1 Behavioural Responses

To test if behavioural emotional contagion was stronger for positive or negative stimuli (crying vs. laughing), and if negative responses were stronger for negative social or non‐social stimuli (crying vs. artificial), we used a linear mixed model (LMM; Baayen 2008) with the mean behavioural response score for each trial (averaged over four 15 s time bins of the stimulus) as the outcome variable, with an interaction between condition (positive, negative, artificial aversive) and site (UK, Budongo, Mbarara) as predictor fixed effects. We further included trial number (one or two), age in weeks, and infant sex (female, male) as control fixed effects, and participant ID as a random intercept. Note that the outcome variable represents the intensity and not the direction of the emotional response, that is, refers to positive reactivity in the laughing condition and negative reactivity in the crying and artificial condition.

#### 2.8.2 Thermal Responses

To account for individual and environmental temperature differences, raw thermal measurements were processed prior to data analysis, by setting nasal temperatures relative to baseline (Kano et al. 2016). We first calculated the mean baseline nasal temperature (based on the three measurements taken during the baseline). Then, we subtracted this mean from each stimulus and recovery measurement to calculate the change in nasal temperature relative to baseline. We included the recovery period immediately following stimulus presentation (30 s), since thermal changes may be temporally delayed and last up to 60 s (Kuraoka and Nakamura 2011). We then further collapsed the data by extracting the maximum change (peaks or troughs) for the first half of the stimulus, the second half of the stimulus, and the recovery period, resulting in three data points per trial. We extracted three measurements, as we were interested if the maximum change would occur at different speed across conditions. All three measurements, including those from the recovery period (because they can be considered to have been elicited during stimulus presentation) were entered into our main analysis.

To test the effect of condition and site on nasal temperature changes, we used a linear mixed model, with maximum temperature change relative to baseline as the outcome variable. As fixed effects, we included an interaction between condition (positive, negative, neutral, artificial aversive) and site (UK, Budongo, Mbarara). We further included measurement time (first half of stimulus, second half of stimulus, recovery), room temperature, trial number (one or two), age in weeks, and infant sex (female, male) as control fixed effects, and participant ID as a random intercept. An additional analysis of the directionality of the thermal response (i.e., temperature increase or decrease by condition) is reported in the Supporting Information S3.

#### 2.8.3 Association Between Thermal and Behavioural Responses

To test if physiological arousal (i.e., thermal responses) was predictive of emotional contagion (i.e., behavioural responses), we used an LMM with the mean behavioural response score for each trial as the outcome variable, and the maximum temperature change per trial (selecting the highest one out of all three timebins) as the predictor fixed effect, additionally entering control fixed effects of condition (positive, negative, artificial aversive), site (UK, Budongo, Mbarara), trial number (one or two), age in weeks, and infant sex (female, male), and participant ID as a random intercept. This model was calculated for a subset of the data not including the babbling condition, as no behavioural responses were coded for this condition (due to no expectation of either aversiveness or emotional contagion for neutral vocalisations).

#### 2.8.4 General Statistical Approach

To establish model significance, we always compared the full model, containing all predictors and interactions of interest, to a null model not containing the two interactions (or the singular predictor of interest for the association between thermal and behavioural responses model) but still containing the control fixed effects and the random intercept, using a likelihood‐ratio test (Dobson 2002). Then, we compared the full model to a main‐effects model into which predictors involved in an interaction in the full model were entered as independent fixed effects without any interactions, to establish significance of the interaction. We used the package lme4 (Bates et al. 2015) to run LMMs, the drop1 function to extract p‐values for individual variables, and the emmeans package (Length 2022) for post‐hoc tests. For model checks, we used variance inflation factors, performed on standard linear models with no interactions and no random effects, which revealed no issues of collinearity. We checked model stability using the functions glmm_stability for binomial GLMMs. To compute confidence intervals, we used bootstrapping with the function bootMer of the package lme4.

## 3 Results

### 3.1 Behavioural Responses

**FIGURE 4:**
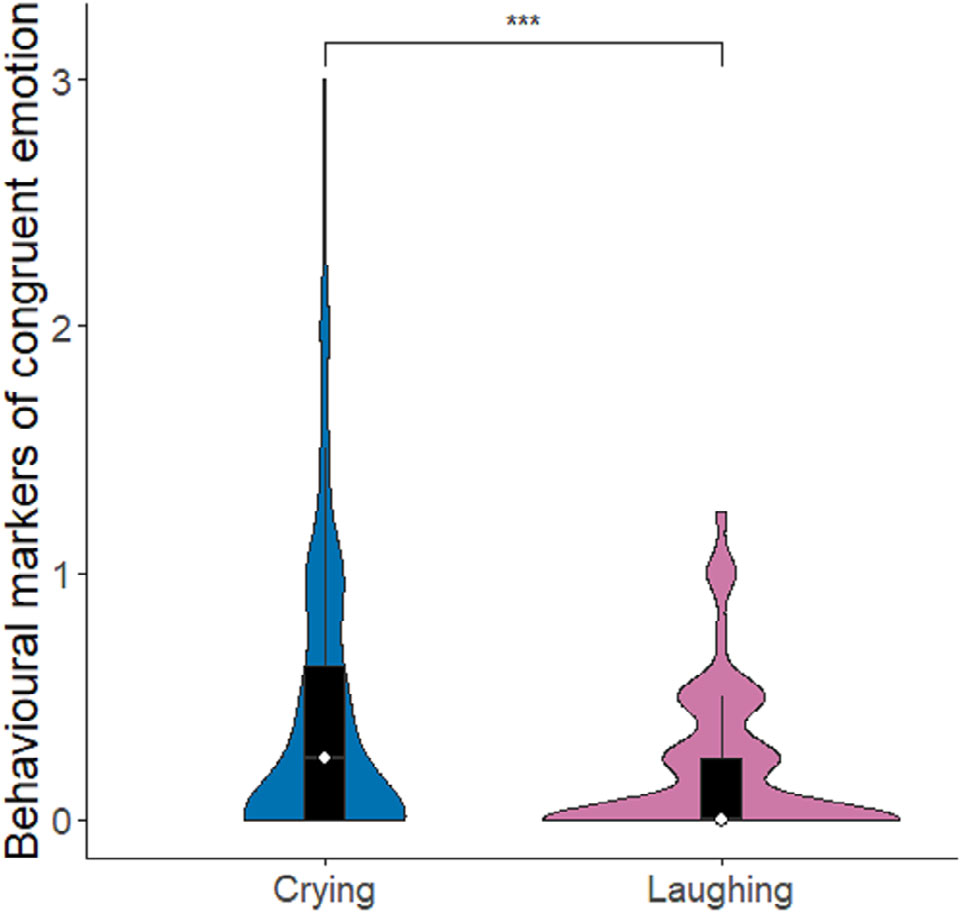
Behavioural markers of emotional contagion in infants in response to playbacks of crying and laughing, combining sites. The rating 0–3 indicates intensity of emotional contagion/congruent emotional markers, that is, negative affect in response to crying and artificial stimuli and positive affect in response to laughing stimuli. Violins show distribution of raw data, the white dot represents the median and the black bar represents the interquartile range. Asterisks represent significant effects (* *p* < 0.05, *** *p* < 0.001).

**FIGURE 5:**
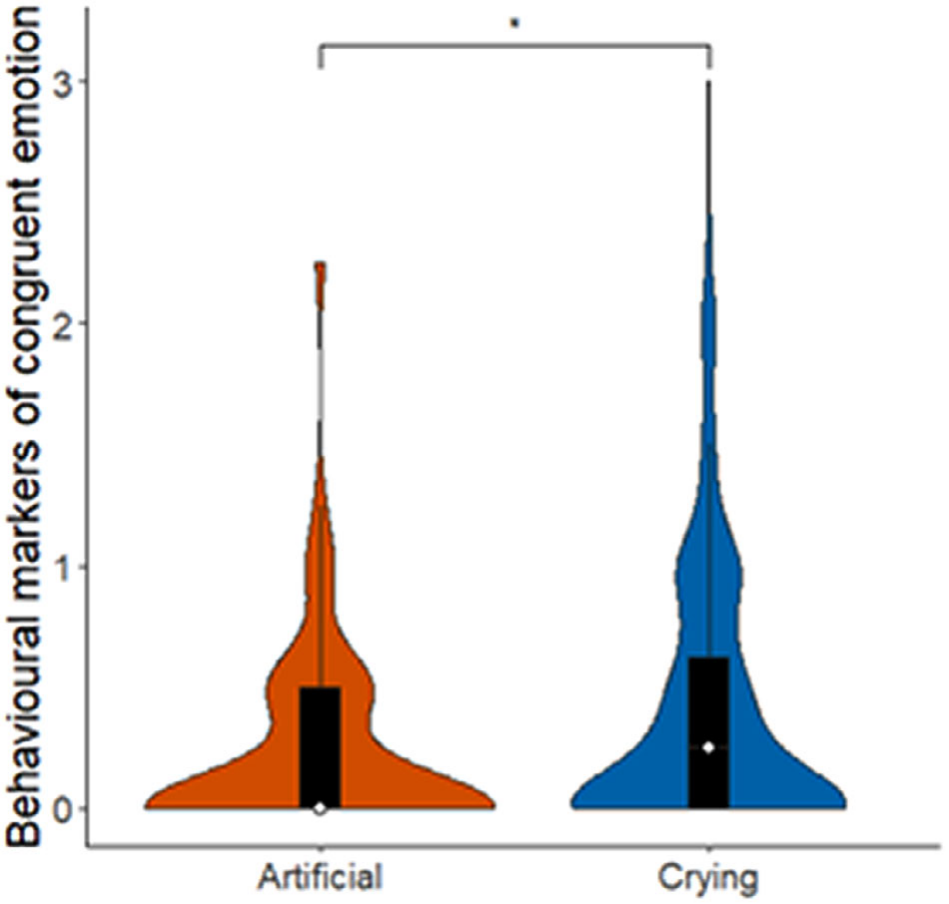
Behavioural markers of emotional contagion in infants in response to playbacks of crying. The rating 0–3 indicates intensity of emotional contagion/congruent emotional markers, that is, negative affect in response to crying and artificial stimuli and positive affect in response to laughing stimuli. Violins show distribution of raw data, the white dot represents the median and the black bar represents the interquartile range. Asterisks represent significant effects (* *p* < 0.05, *** *p* < 0.001) and artificial, combining sites.

The full‐null model comparison was significant (*χ^2^* (8) = 23.44, *p* = 0.002), indicating that condition and site (or their interaction) had a significant effect on infants’ behavioural responses. As the full‐main‐effects model comparison was not significant (*χ^2^* (4) = 2.11, *p* = 0.716), we examined the main‐effects model without the interaction. Using single term deletions, we found that condition, but not site, had a significant effect on infant behavioural responses. Post‐hoc tests revealed that infants showed higher behavioural emotional contagion for crying than for laughing (estimate ± SE = 0.26 ± 0.06, *p* < 0.001, Figure 4), and less negative responses to the aversive artificial stimulus than to crying (estimate ± SE = −0.16 ± 0.06, *p* = 0.034, Figure 5).

### 3.2 Thermal Responses

**FIGURE 6:**
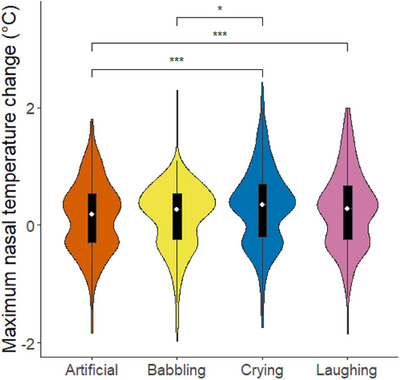
Maximum nose tip temperature change by condition, combining sites. Violins show distribution of raw data, the white dot represents the median and the black bar represents the interquartile range. Asterisks represent significant effects (* *p* < 0.05, *** *p* < 0.001). The rural Ugandan site was Budongo, the urban Ugandan site was Mbarara.

The full‐null model comparison was significant (*χ^2^* (13) = 97.37, *p* < 0.001), indicating that condition and site (or their interaction) had a significant effect on nasal temperature change. However, as the full‐main‐effects model comparison was not significant (*χ^2^* (6) = 10.00, *p* = 0.125) we present the results of the main‐effects model, containing no interaction. Using single term deletions via the drop1 function on the reduced model, we found main effects of condition, site, and measurement time on nasal temperature change. Post‐hoc tests revealed that infant maximum nasal temperature change was smaller for the artificial aversive stimulus than for positive (estimate ± SE = −0.23 ± 0.05, *p* < 0.001) and negative social stimuli (estimate ± SE = −0.24 ± 0.04, *p* < 0.001). We did not find a significant difference between the artificial and neutral stimuli (estimate ± SE = −0.12 ± 0.05, *p* = 0.085). Additionally, temperature changes were smaller in response to neutral stimuli than negative social stimuli (estimate ± SE = −0.12 ± 0.04, *p* = 0.016, see Figure 6). The control effect of measurement time also influenced the thermal response, with the first timebin (the first 30 s of the trial) producing the smallest temperature change compared to the second (estimate ± SE = −0.08 ± 0.03, *p* = 0.024) and third (estimate ± SE = −0.10 ± 0.03, *p* = 0.003) timebins. There was no difference between the second and third timebin, that is, the second half of the stimulus and the recovery period (estimate ± SE = −0.02 ± 0.03, *p* = 0.776). These timebin differences are reflective of the relatively slow nature of thermal responses and show that the response extended into the recovery period.

**FIGURE 7:**
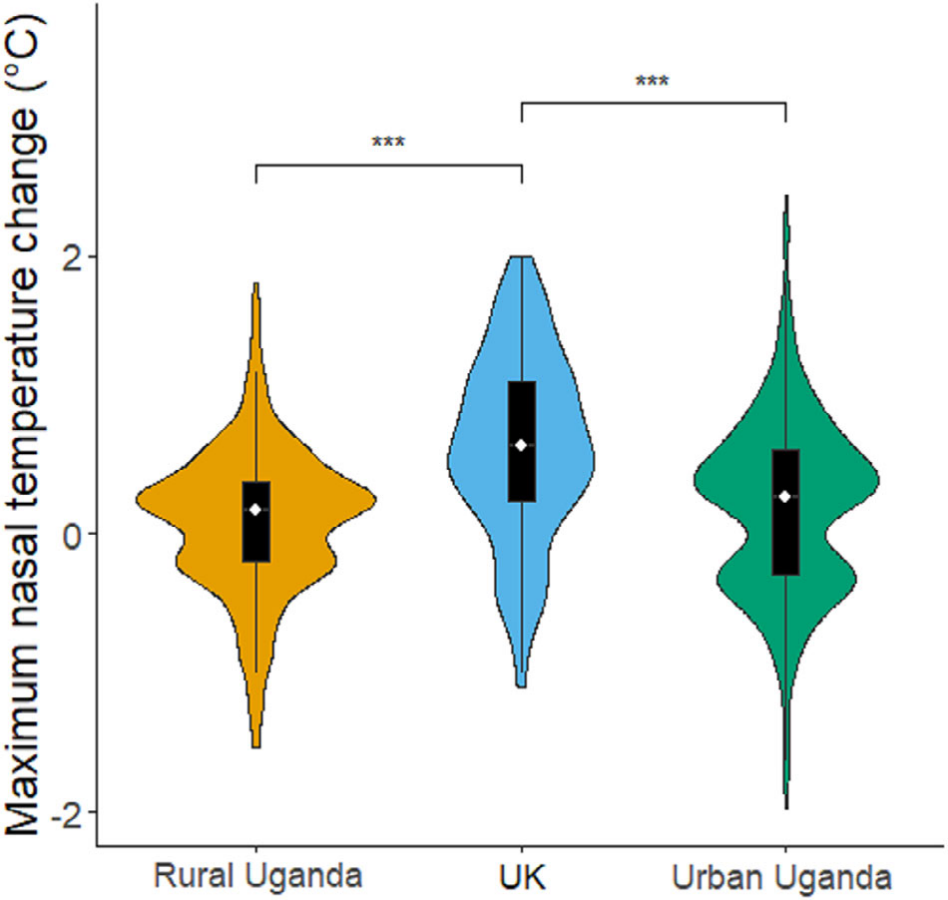
Maximum nose tip temperature change by site, combining Conditions. Violins show distribution of raw data, the white dot represents the median and the black bar represents the interquartile range. Asterisks represent significant effects (* *p* < 0.05, *** *p* < 0.001). The rural Ugandan site was Budongo, the urban Ugandan site was Mbarara.

Overall, temperature changes were significantly higher in the UK sample than either the urban Ugandan sample in Mbarara (estimate ± SE = 0.46 ± 0.08, *p* < 0.001) or the rural Ugandan sample in Budongo (estimate ± SE = 0.56 ± 0.14, *p* < 0.001, see Figure 7). This is unlikely to be due to differences in environmental conditions, since we controlled for room temperature, and this variable was also not significant in our models. See Supporting Information S4 for full model estimates, standard errors, and confidence intervals.

### 3.3 Association Between Thermal and Behavioural Responses

The full‐null model comparison was not significant (*χ^2^* (1) = 0.00, *p* = 0.99), indicating that the maximum thermal change relative to baseline was not predictive of infant behavioural responses to the stimuli.

## 4 Discussion

Previous research has documented contagious crying—the transfer of emotional distress of one infant to another—to be a robust phenomenon during infancy (e.g., Dondi, Simion, and Caltran 1999; Geangu et al. 2010; Simner 1971). This form of emotional contagion has long been considered an early form, or a precursor, of empathic responding, whereby infants experience others’ emotions, but do not yet possess the cognitive and regulatory capacities required for other‐oriented concern, thus resulting instead in their own personal distress (Eisenberg, Fabes, and Spinrad 2006; Preston and de Waal 2002). However, certain limitations in previous research have left some questions unanswered: in particular, a lack of appropriate controls has meant that it has remained unclear whether infant distress in response to peer crying is due to emotional contagion or more simply due to aversive acoustic features of cry vocalisations (Ruffman, Lorimer, and Scarf 2017). Additionally, the use of long, intense stimuli in previous studies may have led to an overestimation of the contagious crying phenomenon, as these stimuli may have simply produced an emotional overarousal and not an emotional contagion response (Davidov et al. 2013). Furthermore, affective state matching can occur without behavioural manifestations (Preston and Hofelich 2012), so research on emotional contagion development needs to extend beyond behavioural measures. Lastly, previous studies on this topic have been heavily biased in their sampling towards infants from Western industrialised contexts, overlooking possible socio‐cultural influences on emotional development.

The aim of the current study was to address these methodological and sampling constraints though a cross‐cultural investigation into emotional contagion in infants using an adaptation of the contagious crying paradigm. Through a combination of infrared thermal imaging and classic behavioural measures, we analysed the physiological and behavioural responses of 10‐ to 11‐month‐old infants from Uganda and the United Kingdom to hearing audio stimuli of infant babbling, laughing, and crying, as well as an artificial stimulus matched to the aversive acoustic features of crying.

In line with our predictions, we found both behavioural and physiological markers of emotional contagion which occurred similarly across all three cultural settings sampled: Infants in two sites in Uganda (one rural and one urban) and one in the United Kingdom showed greater changes in nasal temperature in response to crying than to babbling, as well as to the artificial stimulus. Taken together, these results suggest that infants are responsive to the emotional content of crying, compared to emotionally neutral peer vocalisations, and that this is not just due to the acoustical aversiveness of crying, as argued by Ruffman, Lorimer, and Scarf (2017). Additionally, infants in our study also showed a greater thermal response to hearing infant laughing as compared to the artificial aversive stimulus. Therefore, infants appear to find social stimuli of both positive and negative emotional valence more emotionally arousing than a non‐social stimulus, even if that stimulus is acoustically aversive. While we found greater thermal responses for crying compared to babbling, this effect was not found for laughing compared to babbling. The lack of a significant difference between laughing and babbling could indicate less emotional contagion for positive emotions, hinting at a “negativity bias,” that is, the preferential processing of negative emotional information (Vaish, Grossmann, and Woodward 2008). What we did not find, however, was a difference in thermal changes between crying and laughing. Both laughing and crying are emotional vocalisations which can be assumed to elicit at least some emotional arousal. Babbling on the other hand may be emotionally neutral, and thus less emotionally arousing, making arousal‐differences measurable. Thermal imaging appears to be a measure of emotional arousal but not necessarily valence, similarly to other measures of physiological emotional arousal. Previous work using pupillometry instead of thermal imaging as a physiological marker of emotional arousal has also not shown valence‐based response differences (Bradley et al. 2008; Partala and Suraka 2003; although see Geangu et al. 2011), a pattern which the similarity between the thermal changes in response to crying and laughing observed in this study fits into.

We also examined behavioural responses to the same stimuli and found that infants also showed higher negative affect in response to crying than to the aversive artificial stimulus. Although infants may be responding to the aversive acoustic features of crying (Ruffman, Lorimer, and Scarf 2017), the greater thermal and behavioural reaction to crying than to an acoustically matched non‐emotional stimulus suggests there to also be an additional emotional contagion response related to the social nature of the stimuli. As in our physiological data, we also found a similar trend towards a behavioural negativity bias, with infants showing greater emotional contagion, as evidenced by affective matching of facial expressions, for crying than for laughing. This is in line with Geangu et al. (2011) but contrary to Ruffman et al. (2019) who showed behavioural emotional contagion (happy or sad facial expressions) for laughing but not for crying in 2‐year‐olds.

When considered in parallel, the overall pattern of the behavioural response follows that of the thermal response—stronger responses to crying than to aversive and laughing—but on an individual level they were not linked. Evidence that on a group‐level they follow the same pattern supports the interpretation of thermal changes as a valid marker of emotional arousal in an emotional contagion scenario. There are at least two, not necessarily mutually exclusive, possible explanations for this lack of apparent direct relation between thermal and behavioural measures. First, methodologically, our assessments of behaviour and physiology occur on different temporal and mathematical scales. Thermal responses are temporally slow to occur (Kuraoka and Nakamura 2011), while behavioural responses occur almost immediately. Second, analytically, we used maximum relative changes (to baseline) as our thermal measurement compared to absolute ratings of behavioural affect. Although these are in keeping with established norms for each method, they may mean that the extracted data are not ideally suitable to be linked to one another. This may also be the reason why other studies on thermal imaging in infants and young children have used both physiological and behavioural measures but have not attempted to link them. In this respect, our exploratory finding of a lack of direct link on an individual level between thermal changes and behavioural reactivity does not invalidate thermal imaging as an emotional arousal measurement. Different measures of emotions rarely show strong convergence, suggesting that emotions are multiply determined and multi‐dimensional, with no one measure being able to capture every aspect of them and two measures likely capturing different aspects of one emotion (Mauss and Robinson 2009). Instead, this opens a new avenue for future research. Alternatively, a direct link between physiological arousal and behavioural response could also potentially suggest an intermediary mechanism on the individual level, whereby the initial contagious arousal is down‐regulated through emotion regulation in order to not lead to an automatic negative behavioural response (de Waal and Preston 2017). In adults, emotion regulation strategies have been shown to decrease coherence between physiological and behavioural measures (Dan‐Glauser and Gross 2013), although it is an open question to what extent this effect is present in infancy. On a behavioural level, prior research has shown that infants high in negative reactivity were more empathic later on if they were sufficiently able to regulate their emotions (Abramson et al. 2019). It seems emotion regulation may come into effect even earlier, at the negative reactivity stage. Although methodological explanations should first be addressed and ruled out, future research should also target the precise mechanisms by which physiological emotional contagion can be down‐regulated before it manifests behaviourally.

Infants showed greater reactivity to crying than to the artificial aversive control stimulus, both physiologically and behaviourally. Although both sounds may be acoustically aversive, which explains the arousal response to the artificial sound (Reuter and Oehler 2011), the crying stimuli contained additional emotional content leading to emotional contagion. This contagious arousal then motivates an empathic comforting response from the listener (Preston and de Waal 2002). Alleviation of the other individual's distress then also decreases contagious arousal in oneself. Infants at this age are sensitive to and attempt to comfort others in distress (Davidov et al. 2021; Roth‐Hanania, Davidov, and Zahn‐Waxler 2011; Vreden et al. in prep.), which likely makes the crying stimuli in this study more arousing: because intervention is not possible with a playback stimulus, the infant cannot stop the other's crying to alleviate their own distress.

Previous contagious crying studies have used long and intense stimuli, eliciting high levels of emotional arousal that, due to an inability to regulate this arousal, result in infant self‐distress (e.g., Geangu et al. 2010; Simner 1971). One aim of this study was to use distress stimuli which were suited to the regulatory abilities of infants to examine their ability to cope with emotional contagion in the absence of empathic self‐distress. With our shorter stimuli, behavioural markers of emotional contagion were relatively infrequent in our sample: Only half of crying or laughing trials resulted in an emotional behavioural response. However, infants still did show measurable changes in behaviour, even with strict control conditions for emotional content and acoustic features, implying both positive and negative emotional contagion. The relatively low levels of behavioural emotional contagion thus do not represent an absence of emotional contagion and empathy as others have argued (e.g., Ruffman, Lorimer, and Scarf 2017, Ruffman et al. 2019), but rather show that infants are able to respond to the emotions of others without becoming overwhelmed by them.

Unexpectedly, we found that United Kingdom infants overall had stronger thermal (but not behavioural) responses to the stimuli in all conditions than Ugandan infants from both the rural and the urban site. We found no interaction effects between site and condition and controlled for room temperature, so it is unlikely that this result is either due to a difference in emotion perception or contagion or environmental conditions of the testing locations. The participants’ capacity for thermal responses might have also been affected by their baseline facial temperature. Average temperatures during the baseline phase differed between all three sites, but the stimulus‐response difference only manifested between all three sites (see Supporting Information). Additionally, Chotard, Ioannou, and Davila‐Ross (2018) found no difference between indoor and outdoor testing conditions on facial temperature changes, although it should be noted that this was with nonhuman primates. Therefore, we do not believe the site difference in the present study to be a result of environmental factors. An alternative, social explanation is that Ugandan infants have more regular exposure to peer vocalisations of any emotional valence, due to differences in household size and composition and more distributed caregiving (Holden et al. 2022). In contrast, the United Kingdom infants in our sample were cared for exclusively in the home environment by parents on parental leave and did not attend nurseries. Increased exposure might have thus led to Ugandan infants being more habituated to noise disturbances, including infant signals, and thus correspondingly less physiologically activated (Li et al. 2018). Since the artificial aversive stimulus was acoustically matched to crying, this exposure effect could have transferred.

One limitation of using a novel method like thermal imaging is that, based on our current state of knowledge, the expected directionality of the thermal change is not clear. Some researchers have found that negative stimuli would lead to drops in nasal temperature (e.g., Ioannou et al. 2013; Kano et al. 2016). However, other studies have found temperature increases linked to negative emotional arousal (e.g., Aureli et al. 2015; Ioannou et al. 2016), as well as temperature decreases for positive emotions (Nakanishi and Imai‐Matsumura 2008). In this study, upon visual inspection of the raw data, we found temperature increases and decreases for both positive and negative stimuli (and the directionality of the maximum change was not associated with condition; see Supporting Information S3). When comparing the directionality of change across different studies, it is important to keep in mind that valence (positive vs. negative) is only one dimension of emotional classification. The other dimension to be considered is high versus low activation or arousal (Russell 1980). While sadness, fear, and stress are all negatively valenced, the latter two can be considered higher in activation than sadness, which may explain why they produce decreases in nose tip temperature (Ioannou, Gallese, and Merla 2014), but sadness does not (as evidenced in our study but also e.g., Marqués‐Sánchez et al. 2020). The interplay between valence and activation leading to mixed results with regards to detecting valence‐based differences is reflected in other physiological measures of emotional contagion, such as pupillometry (Bradley et al. 2008). This should not be understood as an invalidation of physiology as a measure but rather as a call for further research to disentangle if and how emotional valence affects the direction of thermal responses, which are primarily activated by emotional arousal, to enable us to use physiological data to measure more than pure emotional arousal of any valence.

Another limitation regards our behavioural coding: Although our coding and that in previous papers, on which our coding scheme was based, have reliably distinguished between expressions of self‐distress and other‐oriented concern, detecting this distinction can be challenging for relatively mild expressions of negative affect. Future studies could address this issue using an application of a more detailed facial expression analysis, such as using facial‐action coding systems that identify muscle movements on a finer scale. This, paired with thermal imaging, could provide an even more fine‐grained and in‐depth look at the physiological underpinnings of emotional contagion and expression.

In summary, by using a novel application of thermal imagery with behavioural measures in a culturally diverse sample, our study addressed both methodological constraints of previous research on emotional contagion in infants and the persistent sampling bias in developmental psychology. We found that 10‐ to 11‐month‐old infants displayed emotional arousal, linked to emotional contagion, on both a behavioural and physiological level, in response to emotional peer vocalisations, and that in the case of crying, this was not simply a product of its aversive acoustic features. We further found a negativity bias in behavioural and thermal responses, with crying eliciting the strongest response behaviourally and physiologically. Our results support the presence of emotional contagion in infancy (e.g., Geangu et al. 2011; Martin and Clark 1982; Simner 1971). This evidence for the early sharing of emotions is fundamental to our understanding of one of the key building blocks in how infants develop the ability to respond to others’ emotions in an empathic manner.

## Conflicts of Interest

The authors declare no conflicts of interest.

## Ethics Statement

Ethical approvals were obtained from the Department of Psychology Ethics Committees at Durham University, the Ugandan Virus Research Institute Regional Ethics Committee (GC/127/20/08/788) and the Ugandan National Council for Science and Technology (SS596ES). Due to low levels of literacy in Uganda, especially in the rural Ugandan sample, local researchers read out the information sheet and consent form in a local language where needed and mothers who could not write consented verbally, accompanied with a thumb print which is standard procedure for signatures in Uganda. In the United Kingdom, written informed consent was obtained through an online platform.

## Supporting information



Supporting Information

## Data Availability

Data will be made available upon publication.
