# Supplementary material for: Contagious Crying Revisited: A Cross‐Cultural Investigation Into Infant Emotion Contagion Using Infrared Thermal Imaging
Source: Dev Sci. 2025 Jan 14;28(2):e13608. doi: 10.1111/desc.13608 (PMC11733258; doi:10.1111/desc.13608)
Supplement: Supplementary file 1 — Supporting Information [file DESC-28-e13608-s001.docx]

# Supplementary Materials

**S1 Stimulus Sources**

The table below contains source URLs from the raw audio files processed into stimuli. Note that the available recordings are not representative of the final stimuli. We thank the lab families who kindly allowed us to use recordings of their children for our stimuli.

| Condition | Source |
| --- | --- |
| Babbling | 395198__bvaudio__baby-babble.mp3 from Freesound.org:  <https://freesound.org/people/bvaudio/sounds/395198/>  Two other stimuli from lab members and one from another researcher. |
| Crying | Three stimuli from lab members and one from another researcher. |
| Laughing | 395491__atcraiger__baby-boy-laughing.wav from Freesound.org:  <https://freesound.org/people/ATCraiger/sounds/395491/>  537312__andreaskg__baby-laughing-around-6-months-old.wav from Freesound.org:  <https://freesound.org/people/andreaskg/sounds/537312/>  One other stimulus from lab members and one from another researcher. |
| Artificial | 395400__wolfercz__siren.wav from Freesound.org:  <https://freesound.org/people/wolferCZ/sounds/395400/>  422051__inspectorj__car-alarm-distant-a.wav from Freesound.org:  <https://freesound.org/people/InspectorJ/sounds/422051/>  383219__kinoton__car-alarm-siren.wav from Freesound.org:  <https://freesound.org/people/Kinoton/sounds/383219/>  479975__craigsmith__r04-22-long-car-horn-blast.wav from Freesound.org:  <https://freesound.org/people/craigsmith/sounds/479975/> |

**S2 Behavioural Coding Scheme**

| Infant Behaviour | Score | Description |
| --- | --- | --- |
| Positive Affect | 0 | No positive affect, neutral face with facial muscles relaxed or other affective facial expression (e.g., negative affect) |
|  | 1 | Low intensity smiling (corners of mouth go up), no accompanying positive vocalisation |
|  | 2 | Medium intensity smiling (corners of mouth go up, cheeks raised), can be paired with chuckling/giggling (but not full open-mouthed laughter) |
|  | 3 | High intensity smile paired with open-mouthed laughing vocalisation |
| Negative Affect | 0 | No negative affect indicating no distress, neutral face with facial muscles relaxed or other affective facial expression (e.g., positive affect) |
|  | 1 | Low intensity negative affect indicating distress including grimacing/frowning (corners of mouth go down), furrowed brows, no accompanying vocalisation |
|  | 2 | Medium intensity negative affect indicating distress including grimacing/frowning paired with whining/whimpering/fussing or shorter cry/protest yell vocalisation of <2s |
|  | 3 | High intensity negative indicating distress open-mouthed continuous crying for >2s |

**S3 Directionality of Thermal Change**

**Thermal Response Directionality**

As previous literature has produced mixed findings on which emotional valence leads to temperature increases or decreases (e.g., Kano et al., 2016; Aureli et al., 2015), we conducted an additional analysis to examine if condition predicted temperature increases or decreases. For each trial, we extracted the maximum change relative to baseline and coded this as increase (greatest change from baseline is a higher temperature than baseline) or decrease (greatest change is a lower temperature). This was entered into a binomial Generalised Linear Mixed Model with direction of change (increase, decrease) as the outcome variable. As fixed effects, we included condition (positive, negative, neutral, artificial aversive) and control fixed effects of site (UK, Budongo, Mbarara), trial number (one or two), age in weeks, and infant sex (female, male), and participant ID as a random intercept.

The full-null model comparison (dropping condition) was not significant (*χ^2^*(3) = 2.41, *p* = .49), indicating that condition was not related to whether nasal temperature increased or decreased relative to baseline.

**S4 Model Estimates for Main Analyses**

**Thermal Responses**

Results of the reduced model of the effects of condition, site, timebin, room temperature, trial number, age, and infant sex on maximum changes in nasal temperature relative to baseline (estimates, together with standard errors, and confidence intervals).

| Term | Estimate | SE | Lower CI | Upper CI | *χ^2^* | df | p |
| --- | --- | --- | --- | --- | --- | --- | --- |
| (Intercept) | -0.096 | 0.594 | -1.222 | 1.046 |  |  | ^(1)^ |
| Condition (Babbling) | 0.122 | 0.052 | 0.018 | 0.216 | 45.748 | 3 | <.001 |
| Condition (Crying) | 0.237 | 0.037 | 0.158 | 0.304 | 45.748 | 3 | <.001 |
| Condition (Laughing) | 0.225 | 0.052 | 0.126 | 0.323 | 45.748 | 3 | <.001 |
| Site (UK) | 0.557 | 0.136 | 0.288 | 0.832 | 30.345 | 2 | <.001 |
| Site (Mbarara) | 0.097 | 0.093 | -0.085 | 0.279 | 30.345 | 2 | <.001 |
| Timebin (Three) | 0.075 | 0.029 | 0.022 | 0.131 | 12.305 | 2 | 0.002 |
| Timebin (Two) | 0.095 | 0.029 | 0.040 | 0.152 | 12.305 | 2 | 0.002 |
| Room Temperature | -0.001 | 0.015 | -0.029 | 0.027 | 0.003 | 1 | 0.958 |
| Trial Number | -0.001 | 0.024 | -0.048 | 0.045 | 0.001 | 1 | 0.975 |
| Age (Weeks) | -0.001 | 0.010 | -0.020 | 0.017 | 0.005 | 1 | 0.942 |
| Sex (Male) | 0.018 | 0.049 | -0.080 | 0.117 | 0.139 | 1 | 0.710 |

^(1)^ Not indicated because of having a very limited interpretation

**Behavioural Responses**

Results of the reduced model of the effects of condition, site, trial number, age, and infant sex on behavioural affective responses (estimates, together with standard errors, and confidence intervals). This model includes only the crying, laughing, and artificial conditions.

| Term | Estimate | SE | Lower CI | Upper CI | *χ^2^* | df | p |
| --- | --- | --- | --- | --- | --- | --- | --- |
| (Intercept) | 0.924 | 0.591 | -0.112 | 2.165 |  |  | ^(1)^ |
| Condition (Crying) | 0.157 | 0.062 | 0.032 | 0.269 | 19.139 | 2 | <.001 |
| Condition (Laughing) | -0.106 | 0.083 | -0.250 | 0.038 | 19.139 | 2 | <.001 |
| Site (UK) | 0.022 | 0.089 | -0.147 | 0.186 | 3.025 | 2 | 0.220 |
| Site (Mbarara) | -0.101 | 0.085 | -0.262 | 0.064 | 3.025 | 2 | 0.220 |
| Trial Number | 0.132 | 0.046 | 0.041 | 0.220 | 8.234 | 1 | 0.004 |
| Age (Weeks) | -0.017 | 0.012 | -0.042 | 0.004 | 2.057 | 1 | 0.151 |
| Sex (Male) | -0.073 | 0.060 | -0.187 | 0.041 | 1.547 | 1 | 0.214 |

^(1)^ Not indicated because of having a very limited interpretation

**Association Between Thermal and Behavioural Responses**

Results of the full model of the effects of the maximum temperature change per trial, condition, site, trial number, age, and infant sex on the mean behavioural response score per trial (estimates, together with standard errors, and confidence intervals).

| Term | Estimate | SE | Lower CI | Upper CI | *χ^2^* | df | p |
| --- | --- | --- | --- | --- | --- | --- | --- |
| (Intercept) | 0.924 | 0.593 | -0.263 | 2.083 |  |  | ^(1)^ |
| Maximum Temperature Change | -0.000 | 0.034 | -0.067 | 0.064 | 0.000 | 1 | 0.990 |
| Condition (Crying) | 0.158 | 0.063 | 0.039 | 0.283 | 19.059 | 2 | <.001 |
| Condition (Laughing) | -0.106 | 0.083 | -0.270 | 0.062 | 19.059 | 2 | <.001 |
| Site (UK) | 0.022 | 0.094 | -0.150 | 0.208 | 2.882 | 2 | 0.237 |
| Site (Mbarara) | -0.100 | 0.085 | -0.264 | 0.083 | 2.882 | 2 | 0.237 |
| Trial Number | 0.132 | 0.046 | 0.037 | 0.218 | 0.004 | 1 | 0.004 |
| Age (Weeks) | -0.017 | 0.012 | -0.040 | 0.007 | 0.152 | 1 | 0.152 |
| Sex (Male) | -0.073 | 0.060 | -0.186 | 0.042 | 0.213 | 1 | 0.214 |

^(1)^ Not indicated because of having a very limited interpretation

**S5 Analysis of Baseline Nose Tip and Room Temperature by Site**

**Baseline Nose Tip**

We performed a Kruskal-Wallis Test on the temperature of the infant’s nose at the beginning of the trial (based on the first thermal measurement). Baseline temperature differed across sites H(2) = 534.72, p < .001. Pairwise Dunn comparisons revealed significant differences between all three sites. See table below for mean nose tip temperature during the baseline, as well as Dunn test results.

| Site | M (Degrees Celsius) | SD |
| --- | --- | --- |
| Budongo | 35.2 | 1.17 |
| Mbarara | 34.2 | 1.54 |
| UK | 30.5 | 2.92 |
| Comparison | Z | p |
| Budongo – Mbarara | 9.45 | < .001 |
| Budongo – UK | 22.11 | < .001 |
| Mbarara – UK | 18.70 | < .001 |

**Room Temperature**

We performed a Kruskal-Wallis Test on the room temperature at the beginning of each trial. Room temperature differed across sites H(2) = 936.83, p < .001. Pairwise Dunn comparisons revealed significant differences between all three sites. See table below for mean room temperature, as well as Dunn test results.

| Site | M (Degrees Celsius) | SD |
| --- | --- | --- |
| Budongo | 27.5 | 1.44 |
| Mbarara | 23.5 | 1.58 |
| UK | 20.3 | 2.03 |
| Comparison | Z | p |
| Budongo – Mbarara | 21.11 | < .001 |
| Budongo – UK | 30.57 | < .001 |
| Mbarara – UK | 17.31 | < .001 |
